# Correlation analysis of angles κ and α with the refraction and anterior segment parameters in children

**DOI:** 10.1186/s12886-024-03409-6

**Published:** 2024-03-28

**Authors:** Yuhao Ye, Yu Zhao, Zhe Zhang, Ruoyan Wei, Yiyong Xian, Yangyi Huang, Fang Liu, Ye Xu, Xingtao Zhou

**Affiliations:** 1 Department of Ophthalmology and Optometry, Eye and ENT Hospital of Fudan University, Shanghai, China; 2 NHC Key Laboratory of Myopia (Fudan University), Laboratory of Myopia, Chinese Academy of Medical Sciences, Shanghai, China; 3 Shanghai Research Center of Ophthalmology and Optometry, Shanghai, China; 4 Shanghai Engineering Research Center of Laser and Autostereoscopic 3D for Vision Care (20DZ2255000), Shanghai, China

**Keywords:** Angle κ, Angle α, Axial length, Refraction, Keratometry

## Abstract

**Aim:**

To investigate the correlation of angles α and κ with the refractive and biological parameters in children.

**Methods:**

This case-series study included 438 eyes of 219 children (males/females = 105/114, age: 3–15 years). Ocular biometric parameters, including axial length, corneal radius of curvature (CR), white-to-white distance (WTW), angle κ and angle α, were measured using IOL Master 700; auto-refraction were assessed under cycloplegia. The eyes were assigned to different groups based on CR, WTW, and gender to compare the angles α and κ, and analyze the correlations between the differences of biological parameters on angles α and κ.

**Results:**

The means of axial length, CR, WTW, angle α, and angle κ were 23.24 ± 1.14 mm, 7.79 ± 0.27 mm, 11.68 ± 0.41 mm, 0.45 ± 0.25 mm, and 0.27 ± 0.22 mm, respectively. Angle α was correlated with CR and WTW (fixed effect coefficient [FEC] = 0.237, *p* = 0.015; FEC = -0.109, *p* = 0.003; respectively), and angle κ also correlated with CR and WTW (FEC = 0.271, p = 0.003; FEC = -0.147, *p* < 0.001, respectively). Comparing subgroups, the large CR and small WTW group had larger angles α (0.49 ± 0.27 vs. 0.41 ± 0.21, p < 0.001; 0.46 ± 0.27 vs. 0.44 ± 0.21, *p* < 0.05, respectively) and κ (0.29 ± 0.25 vs. 0.24 ± 0.15, *p* < 0.01; 0.29 ± 0.25 vs. 0.26 ± 0.19, p < 0.05, respectively). The differences in interocular angles α and κ showed correlation with interocular WTW (r = − 0.255, *p* < 0.001; r = − 0.385, p < 0.001). Eyes with smaller WTW tended to have larger angle κ (0.28 ± 0.27 vs. 0.25 ± 0.15, *p* < 0.05).

**Conclusion:**

The size of angle α/κ may be correlated to CR and WTW, and a larger WTW eye may suggest a smaller angle κ compared with the fellow eye.

## Background

Investigation on refractive status in children has long been the research hotspot as myopia prevention and control being a worldwide problem. More than 12.8 million (0.96%) children aged 5–15 years are affected by ametropia all over the world and the prevalence of myopia in China remains high [1]. The children are at a higher risk of myopia progression during the COVID-19 pandemic [2–4], and the applications of low-concentration atropine [5] and corneal contact lenses [6] (multifocal contact lenses [MFCL] and orthokeratology [OK]) have shown to be effective in controlling myopia progression. During the stage of emmetropization and ocular growth in childhood, different biological parameters may correlate to refractive parameters, and affect the outcome of clinical prescription.

The angles α and κ significantly affect the incidence of glare and halo in post-cataract surgery patients [7]; the angle κ is also related to surgery induced astigmatism [8] and higher-order aberrations [9] after refractive surgeries in patients with myopia or hyperopia. In the design of scleral contact lenses, the angle κ affects the degree of difference between the nasal and temporal scleral curvatures [10]. Moreover, the size of angle κ significantly affects the evaluation of strabismus and the outcome of the surgery [11]. Studies conducted in adults have shown that the angle κ does not change significantly with age [12] and is comparable between genders [12]. However, the distributions of the sizes of angles α and κ in children remain unclear. Therefore, a study on the distributions of the sizes of angles α and κ and their correlations with the refraction and anterior segment parameters in children will help further elucidate the refractive characteristics of this population and guide the control strategy and treatment of ametropia for children, especially myopia and strabismus.

## Methods

### Study population

This case series study was conducted in accordance with the Declaration of Helsinki. This study was approved by the Ethics Committee of the Eye, Ear, Nose, and Throat Hospital of Fudan University (approval no. 2020022), and a written informed consent was obtained from all subjects. A total of 438 eyes of 219 subjects (males: 105, females: 114) were included in this study. These subjects underwent relevant eye examinations from April to September 2021 at the Eye, Ear, Nose, and Throat Hospital of Fudan University. The inclusion criteria were: 1. subjects aged 3–15 years; 2. subjects with no history of contact lens wear; 3. a slit-lamp examination of the anterior segment and fundus of the eye suggested that the patient had no ocular inflammation, trauma, cataract, glaucoma, or other ocular diseases, and the patient had no contraindications for pupil dilation and no history of systemic diseases.

### Measurements

The patient underwent eye examinations, including the measurements of axial length, corneal radius of curvature (CR), white-to-white distance (WTW), angle κ (Chang-Waring, CW) chord: distance and location of the center of the corneal reflection relative to the center of the pupil), angle α (coordinates of the center of the corneal reflection relative to the center of the corneal fundus), anterior chamber depth (ACD), and anterior aqueous depth (AQD). After performing the measurements, tropicamide was administered (5 times, 5 min each time), and synthetical refraction measurement for parameters including refraction sphere (RS), refraction cylinder (RC), corrected distance visual acuity (CDVA) was performed after another 30 min with complete cycloplegia. Biological parameters were measured by an experienced physician (Y.Y.) using IOL Master 700 (Carl Zeiss AG, Germany). The post-dilatation refraction was measured by another experienced physician (Y.X.) using Auto Ref/Keratometer (ARK-1, Nidek, Co., Japan). The IOL master 700 measurements from each examination were evaluated by a visual fixation assessment to exclude the measurements obtained under visual fixation instability. The time interval between any two measurements were at least 3 minutes to avoid fixation fatigue. Six measurements of quality ≥7/9 were taken in each eye, and the mean value of each parameter was calculated and used as the final value for further analysis.

### Data analysis

The data were analyzed using SPSS software (version 25.0, SPSS, Inc., Chicago, IL, USA). The data were expressed as mean ± standard deviation. The normality of each dataset was assessed using the Shapiro–Wilk test. Repeated measures were analyzed and compared. A multilevel correlation analysis was performed using the mixed effects model to control for the effects of binocular enrollment and the correlation between parameters. The subjects were grouped based on gender, CR, and WTW to compare the differences of angles α and κ between groups. Subjects with higher CR/WTW eyes and those with lower values of the relative parameters were assigned to two different groups to compare the inter-eye differences. Spearman’s correlation analysis was used to study the correlations of the differences in the parameters between the fellow eyes. For data that conformed to a normal distribution, a generalized estimating equation (GEE) was used to test for independent differences between groups, including left and right eye hierarchies. A *p* < 0.05 was considered statistically significant.

## Results

**Fig. 1:**
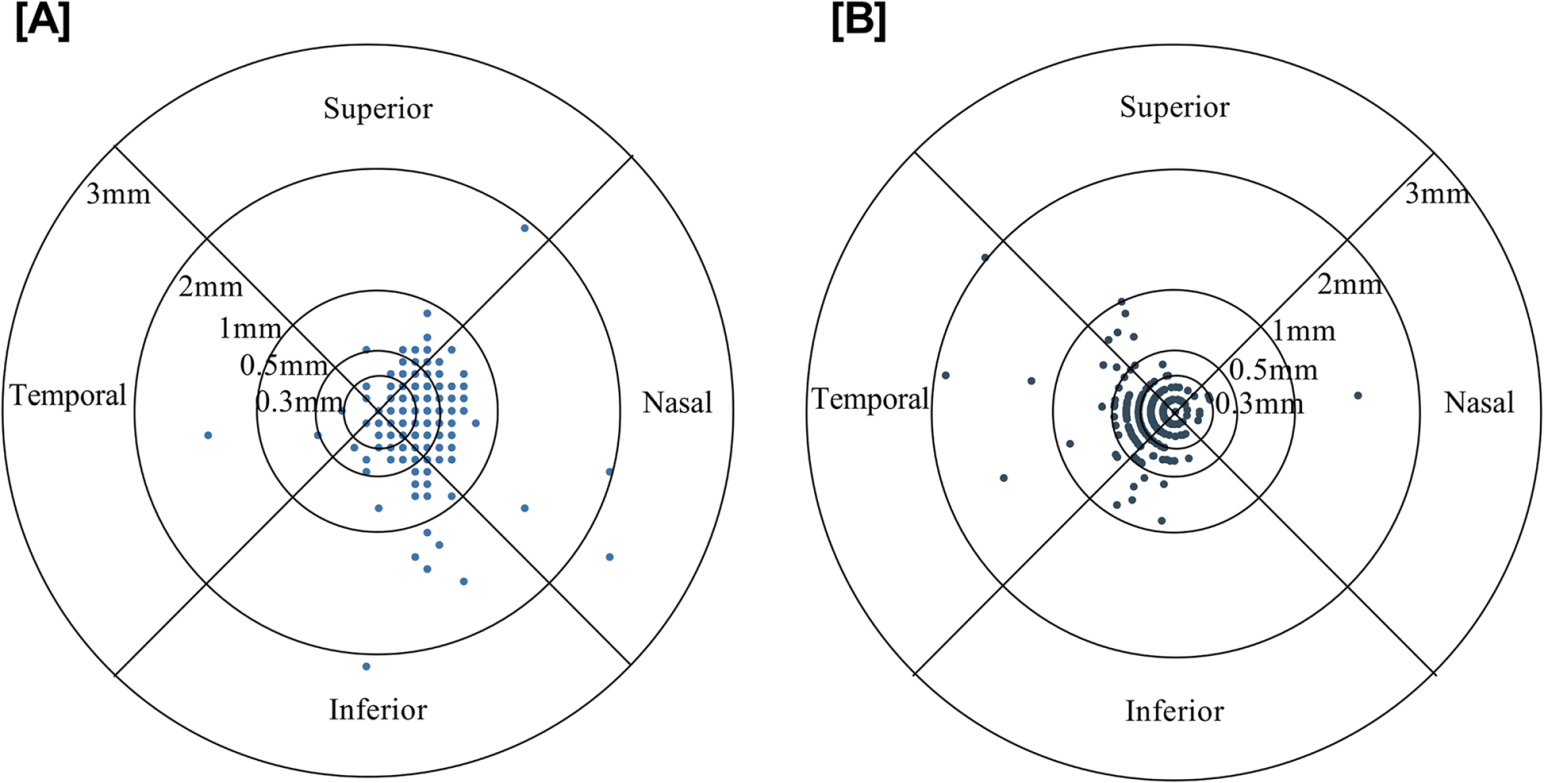
Distribution of angle α **A** and angle κ **B**

Table 1 illustrates the subjects’ characteristics. The mean coordinates of angle α were (0.36, − 0.01) (inferior nasal). The mean coordinates of angle κ were (− 0.20, − 0.03) (inferior temporal). Figure 1 shows the distributions of angles α (Fig. 1A) and κ (Fig. 1B). Table 2 shows the percentages of angles α and κ distributed in different quadrants. There were 6.15, 20.57, 63.13, and 97.40% of the angles α falling within the ranges of 0.2, 0.3, 0.5, and 1.0 mm, respectively. In a previous study, the angles κ were identified as low, medium, and high using 0.3 mm and 0.5 mm as thresholds [13]. In this study, 55.32, 79.67, 94.80, and 98.82% of the angles κ fall within 0.2 mm, low, medium, and high ranges, respectively. The males had flatter corneal curvature (7.90 ± 0.28 vs. 7.69 ± 0.23, *p* < 0.001) and larger WTW (12.34 ± 0.43 vs. 12.14 ± 0.44, p < 0.001) compared to the females.

**Table 1:** The clinical parameters and biometric values of the eyes

Characteristic	Mean ± SD	Range
Age (years)	7.24 ± 2.08	3 to 15
Gender (male/female)	105/114	
Axial length (mm)	23.24 ± 1.14	20.49 to 26.89
Refraction sphere (D)	0.39 ± 1.98	−7.50 to 8.50
Refraction cylinder (D)	−0.69 ± 0.66	−3.50 to 0
Spherical equivalent, SE (D)	0.05 ± 1.98	−8.38 to 7.38
CR-mean(mm)	7.79 ± 0.27	7.06 to 8.81
CCT (mm)	0.52 ± 0.31	0.460 to 0.590
angle α (mm)	0.45 ± 0.25	0 to 2.25
angle κ (mm)	0.27 ± 0.22	0 to 2.00
ACD (mm)	3.61 ± 0.29	2.83 to 4.31
AQD (mm)	3.06 ± 0.28	2.35 to 3.78
WTW (mm)	11.68 ± 0.41	11 to 13

*RS* Refraction sphere, *RC* Refraction cylinder, *SE* spherical equivalent, *CCT* central corneal thickness, *CR* corneal radius of curvatures, *ACD*, anterior chamber depth, *AQD* anterior aqueous depth, *WTW* white to white

**Table 2:** Distribution of percentage of angle α and angle κ in different quadrants

Quadrants	Temporal		Vertical axis		Nasal	
	angle α	angle κ	angle α	angle κ	angle α	angle κ
Superior	1.65%	**37.12%**	0.47%	0.00%	**41.13%**	4.26%
Horizontal axis	0.47%	1.42%	0.24%	3.78%	**20.33%**	0.00%
Inferior	0.95%	**50.83%**	0.71%	0.24%	**34.04%**	2.36%

Values lager than 20% are shown in bold

### Correlation analysis

Table 3 shows the correlation and the corresponding *p*-value for ocular biological or refractive parameters with either angle α or angle κ in the mixed effects model. CR was correlated with both angles α (fixed effect coefficient, FEC = 0.237, *p* = 0.015) and κ (FEC = 0.271, *p* = 0.003). WTW also showed correlations with angles α (FEC = -0.109, p = 0.003) and κ (FEC = -0.147, *p* < 0.001). Other parameters, such as age, gender, ocular axial length, anterior depth, and refractive parameters showed no correlation with angle α or angle κ (*p* > 0.05).

**Table 3:** Association between angle α and angle κ with other factors analyzed with mixed effects model

Factors	angle α		angle κ	
	R (Fixed effect)	P	R (Fixed effect)	P
**CR-mean** **WTW**	**0.237**	**0.015**	**0.271**	**0.003**
	**−0.109**	**0.003**	**−0.147**	**< 0.001**
Age	0	0.915	−0.002	0.818
Gender	0.005	0.837	−0.038	0.107
AL	−0.010	0.792	−0.036	0.286
ACD	−1.022	0.175	−0.519	0.435
AQD	1.023	0.175	0.674	0.312
CCT	0	0.263	0	0.541
RS	0.021	0.177	−0.004	0.767
RC	− 0.029	0.136	− 0.016	0.355

*CCT* central corneal thickness, *CR* corneal radius of curvatures, ACD, anterior chamber depth; AQD, anterior aqueous depth; WTW, white to white; AL: axial length; RS, Refraction sphere; RC, Refraction cylinder. Values with statistical significance are shown in bold

### Intra-group comparison

**Fig. 2:**
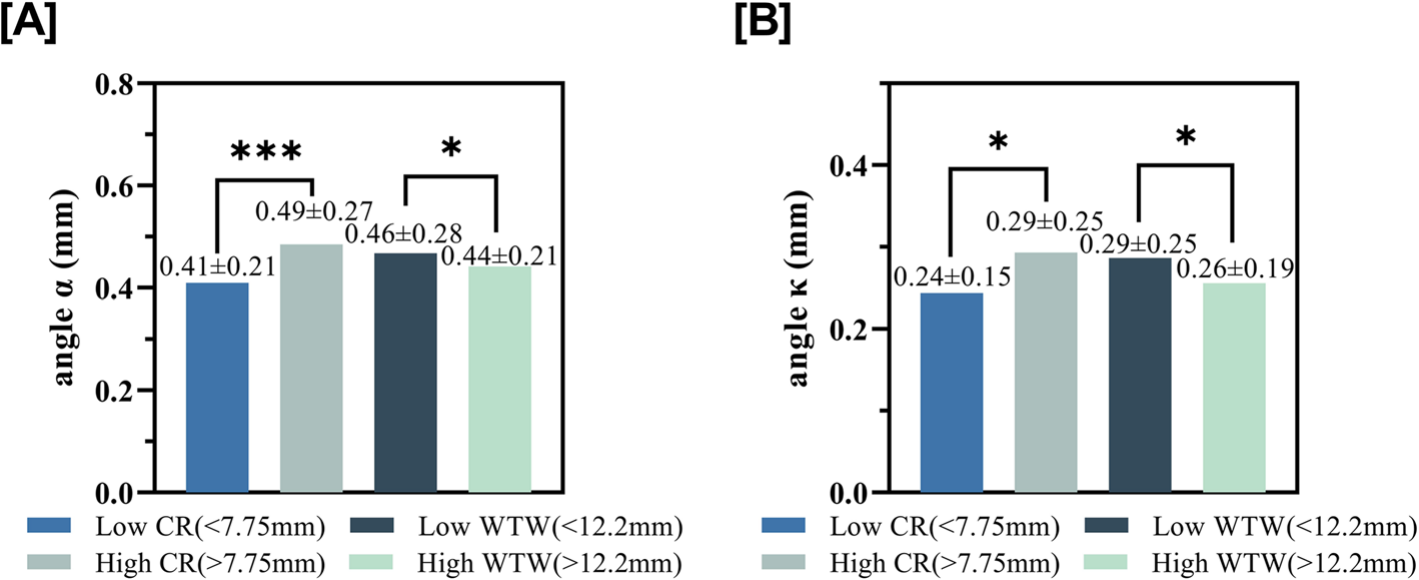
Comparison of angle α **A** and angle κ **B** according to various grouping parameters. CR: corneal radius of curvature, WTW: white to white

**Fig. 3:**
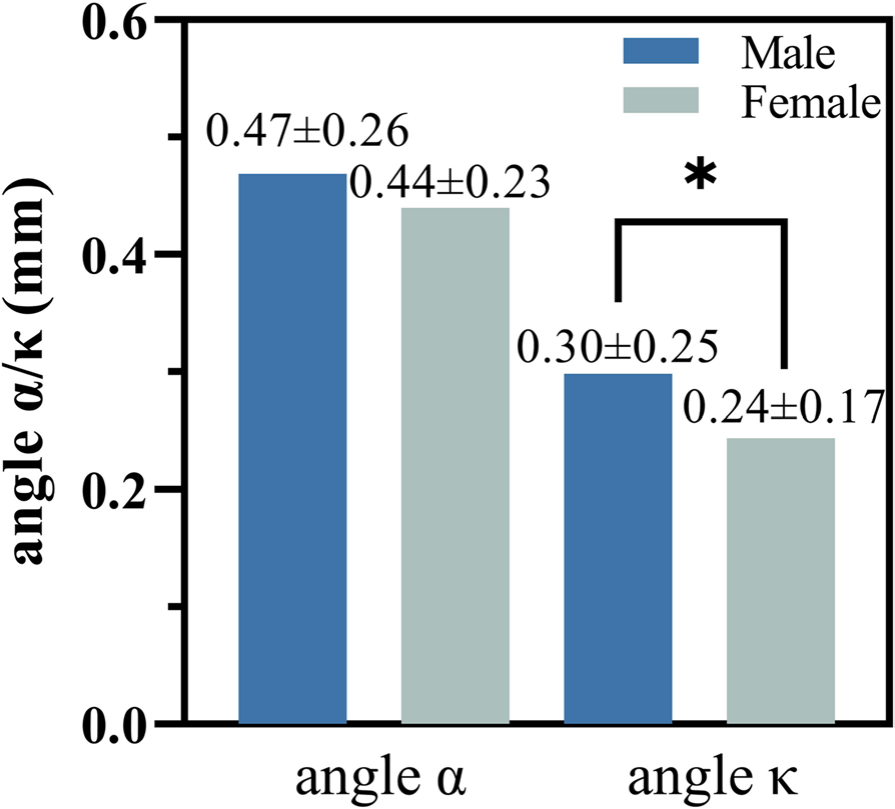
Angle α and angle κ in two different groups based on different genders

The samples were categorized based on CR, WTW, and gender; intra-group comparisons were performed. High CR group (CR > 7.75 mm) and low WTW group (WTW < 12.2 mm) had larger angles α and κ compared to the low CR and high WTW group (Fig. 2). Males tended to have larger angle κ (Fig. 3).

### Comparison between fellow eyes

**Fig. 4:**
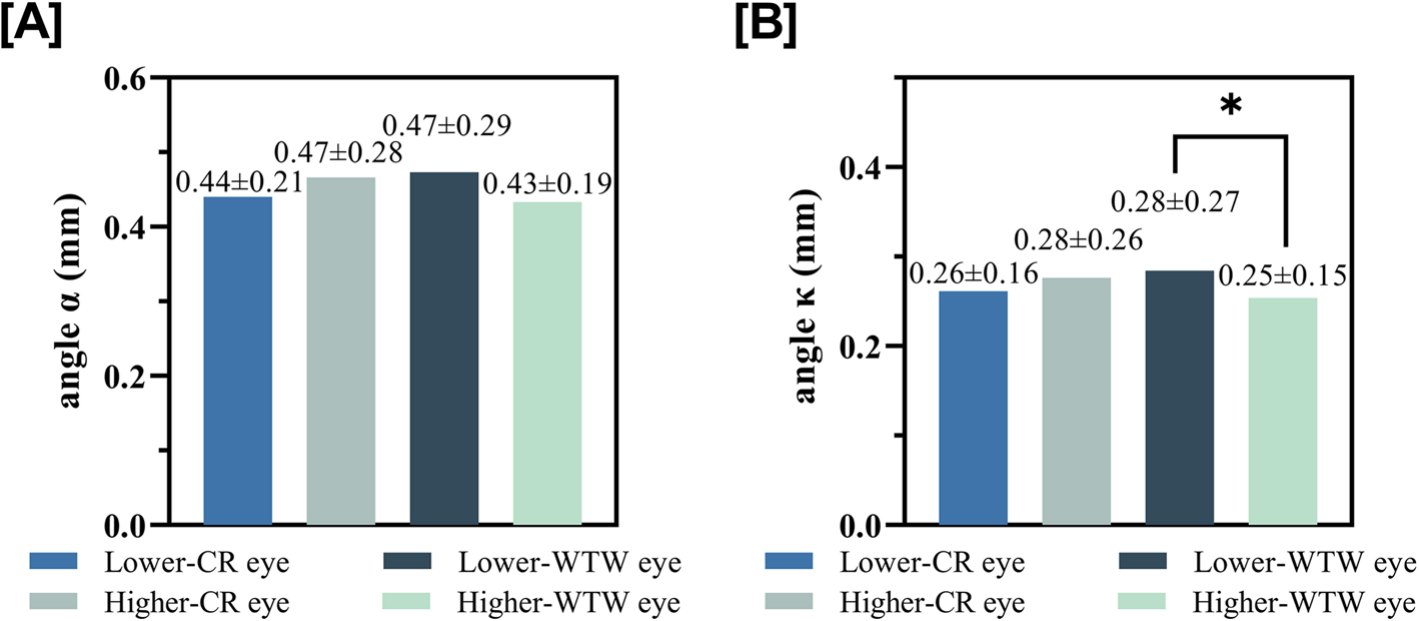
Angle α and angle κ in groups stratified by binocular CR (corneal radius of curvature) or WTW (white to white) differences

Table 4 shows the correlations between the interocular differences of angles α and κ and that of other parameters. The interocular differences of WTW correlated with that of angles α (r = − 0.255, *p* < 0.001) and κ (r = − 0.385, p < 0.001). Figure 4 demonstrates the effects of interocular differences in WTW on the angle κ, with the eye with lower WTW tending to have a larger angle κ compared to the contralateral eye with higher WTW.

**Table 4:** Association between binocular difference of angle α and angle κ with that of other parameters in Spearman correlation analysis

Factors	Δ angle α		Δ angle κ	
	r	P	r	P
Δ CR-mean	0.017	0.821	−0.022	0.775
Δ **WTW**	**−0.255**	**< 0.001**	**−0.385**	**< 0.001**
Δ AL	−0.008	0.905	−0.029	0.681
Δ ACD	0.008	0.906	−0.037	0.599
Δ AQD	0.017	0.808	−0.021	0.768
Δ CCT	−0.040	0.572	−0.006	0.927
Δ RS	−0.001	0.993	−0.001	0.933
Δ RC	0.029	0.683	−0.086	0.231

*CCT* central corneal thickness, *CR* corneal radius of curvatures, *ACD* anterior chamber depth, *AQD* anterior aqueous depth, *WTW* white to white, *AL* axial length, *RS* Refraction sphere, *RC* Refraction cylinder. Values with statistical significance are shown in bold

## Discussion

In the treatment for different vision impairments, such as refractive errors, strabismus, and cataract, the angles α or angle κ can both influence the course and outcome [11, 14, 15].

The angle κ, also known as angle λ, is defined as the angle between the pupillary axis (the line that passes perpendicularly through the center of the pupil and the center of curvature of the cornea) and the visual axis (the line that connects the fixation point with the fovea) [16]. Angle κ was predominantly situated towards the nasal side in myopic subjects aged 16–51 years in Thailand (97%) [17], whereas the finding in this study was diametrically opposite (89.37%). Besides the difference in ages (7.24 ± 2.08 vs. 31.66 ± 7.77 years) between the two studies, the refraction status may be one of the important factors (0.05 ± 1.98 D vs. -4.9 ± 2.29 D). Previous studies have shown that angle κ is correlated with horizontal coma, and there may be a mechanism in the posterior surface of the cornea or the lens to balance the horizontal coma on the anterior surface of the cornea [18, 19]. The angle κ situated towards the temporal side in contrast to the norm during emmetropization may affect the strabismus surgery design [11]. A large angle κ may correspond to a smaller radius of scleral curvature (steeper sclera) in the design of scleral lenses; however, the effect of the quadrant distribution of angle κ on the radius of scleral curvature has not been discussed [10]. Our study revealed that angle κ in children is predominantly distributed inferotemporally, followed by superotemporally. This may influence the design of corneal contact lenses, such as OK lenses, and strabismus surgery. The angle κ-corrected corneal topography can help in the distinguishment of pseudo-deviation after orthokeratology treatment, and the accurate measurement of angle κ is crucial in determination of the actual strabismus angle. The details require elucidation in future studies on specific populations.

Publication concluded angle κ correlates with the axial length (Pearson’s r = − 0.813, *p* < 0.001) and spherical equivalent correlation (SER) (Pearson’s r = 0.685, *p* = 0.003) [10]. However, no correlations between these two parameters and angle κ were found in this study. There were three possible reasons for the differences when compared with previous differences: first, we included children aged 3–15 years, while the other related study included adults aged 24–54 years, and angle κ may vary between children and adults; second, a larger sample size was included in this study (219 vs. 24), which reduces the appearance of false positive results; and third, a mixed-effects model was used in this study to conduct the analysis, which controls for the interactions between hierarchical clustered factors.

It was found that male corneas with flatter corneas (larger CRs) and larger WTW corresponded to larger angles κ. We used GEE to eliminate the hierarchical clustered factors. Gender may be an independent factor influencing angles κ, and comparisons based on WTWs need to be conducted under gender grouping for better comparison of angles κ. A previous study on the 14–81 year-old population found no significant gender differences [16]. Therefore, age may be the main influencing factor. The development of the axial length and corneal curvature differs between males and females [20]. Thus, the findings of this study, which focused on children, differed from the findings of previous studies conducted on adults.

The interocular comparison showed that the differences in interocular WTWs affected the interocular angles κ. Comparing the fellow eyes, we eliminated the possible influencing factors in angle κ between individuals. Differences in interocular WTWs may affect the visual pathways of children, resulting in differences in angle κ. Therefore, in the treatment of congenital cataracts, strabismus, or refractive errors, the effect of differences should also be taken into account on treatment strategies and outcomes between the fellow eyes in pediatric patient.

Previous studies of angle α predominantly focused on the cataract population because it is a more stable pre- and post-cataract surgery measurement compared to angle κ [15, 21]. This study showed that flatter corneas with smaller WTWs had larger angles α, which is similar to the findings on angles κ and consistent with the relationship between angles α and WTW reported in the cataract population [22]. We also found that angle α predominantly oriented towards the nasal side of the optic axis; angle α in the cataract population was mainly situated towards the temporal side of the visual axis (nasal side of the optic axis), and Angle α in the cataract population was shown to decrease non-linearly and shift towards the nasal side of the visual axis (the temporal side of the optic axis) as the axial length increased [15]. The mean axial length in this study was 23.24 ± 1.14 mm; thus, angle α predominantly distributed on the nasal side of the optic axis. Angle α in both the populations showed similar characteristics. Currently, studies on the role of angle α in the treatment of congenital cataracts, strabismus, and refractive errors are scarce, which needs to be addressed with further studies.

The current study has the following limitation. Firstly, we did not standardize for different levels of accommodation in subjects, as previous studies have shown that angles κ are not significantly different between subjects with different levels of accommodation [23]. Secondly, the effect of conditioning on angle κ may be different in children, rendering the other limitation of this study.

## Conclusions

This study firstly describes the distribution of angles α and κ and their correlations with the anterior segment parameters in Shanghai children. In particular, males tend to have larger angles κ; the size of angle α/κ may be related to CR and WTW; a larger WTW may suggest a smaller angle κ in the same patient. This study will help further confirm the refractive characteristics in this population, thus guiding the control and treatment of refractive errors, especially myopia and strabismus.

## Data Availability

The datasets generated and/or analyzed during the current study are not publicly available due to funding requirements but are available from the corresponding author upon reasonable request.

## Abbreviations

CCT: Central corneal thickness
CR: Corneal radius of curvatures
ACD: Anterior chamber depth
AQD: Anterior aqueous depth
WTW: White to white
AL: Axial length
RS: Refraction sphere
RC: Refraction cylinder
CDVA: Corrected distance visual acuity
GEE: Generalized estimating equation
